# A role for *Vibrio vulnificus* PecS during hypoxia

**DOI:** 10.1038/s41598-019-39095-4

**Published:** 2019-02-26

**Authors:** Nabanita Bhattacharyya, Tiffany L. Lemon, Anne Grove

**Affiliations:** 1 Department of Biological Sciences, Louisiana State University, Baton Rouge, Louisiana 70803 USA; 2 Present Address: Department of Epidemiology, Harvard T. H. Chan School of Public Health, Boston, MA USA

## Abstract

The genus *Vibrio* includes serious human pathogens, and mollusks are a significant reservoir for species such as *V*. *vulnificus*. *Vibrio* species encode PecS, a member of the multiple antibiotic resistance regulator (MarR) family of transcription factors; *pecS* is divergently oriented to *pecM*, which encodes an efflux pump. We report here that *Vibrio* species feature frequent duplications of *pecS-pecM* genes, suggesting evolutionary pressures to respond to distinct environmental situations. The single *V*. *vulnificus* PecS binds two sites within the *pecS*-*pecM* intergenic region with K_d_ = 0.3 ± 0.1 nM, a binding that is attenuated by the ligands xanthine and urate, except when promoter DNA is saturated with PecS. A unique target is found in the intergenic region between genes encoding the nitric oxide sensing transcription factor, NsrR, and *nod*; the *nod*-encoded nitric oxide dioxygenase is important for preventing nitric oxide stress. Reporter gene assays show that PecS-mediated repression of gene expression can be relieved in presence of ligand. Since xanthine and urate are produced as part of the oxidative burst during host defenses and under molluscan hypoxia, we propose that these intermediates in the host purine degradation pathway function to promote bacterial survival during hypoxia and oxidative stress.

## Introduction

*Vibrio vulnificus* is a Gram-negative bacterium that inhabits estuarine environments. The bacteria prefer a growth temperature of 22–30 °C and a salinity of 15–25 ppt, and they are therefore more abundant in waters from the warmer Gulf Coast than the cooler New England and Pacific coasts^1^. *V*. *vulnificus*-related gastroenteritis infections are commonly known to be caused by the consumption of raw or undercooked seafood such as oysters^2^. Filter feeders such as oysters, clams, mussels, and scallops concentrate bacteria that are associated with plankton, thereby acting as passive carriers to allow the entry of Vibrios into the food chain and infecting humans^3^. Strains of *V*. *vulnificus* can survive in oysters under cold storage and certain encapsulated strains can survive the phagocytic effects of hemocytes.

Mollusks are an important ecological niche for Vibrios. Since the animals generally have an open circulatory system, the bacteria encounter hemolymph components during the filter-feeding process. Such components include hemocyanin, which is used for oxygen transport^4^. Hemocyanin’s ability to bind oxygen depends on environmental conditions, and oxygen binding is increased by allosteric binding of naturally occurring metabolites such as urate, which accumulates during hypoxia^5^. Urate is the product of xanthine oxidoreductase, which participates in purine salvage and degradation by converting hypoxanthine to xanthine and xanthine to urate; urate accumulates during hypoxia because degradation of urate is an oxygen-dependent process^6,7^. Accordingly, xanthine and urate would also be among the hemolymph components encountered by Vibrios, particularly when the mollusks experience hypoxia. Marine ecosystems – and resident sessile species such as mollusks – frequently experience hypoxia^8^. Under hypoxia and elevated CO_2_ levels **(**hypercapnia), oysters also have a decreased ability to inactivate bacteria within their tissues compared to oysters maintained under normoxic conditions. As a result, oysters that experience hypoxia and hypercapnia retain a larger number of culturable bacteria^9^.

The generation of reactive oxygen species (ROS) by a host (including mollusk) in response to invading bacteria is a first attempt at killing the invader; ROS will react with bacterial macromolecules to cause injury or death unless the ROS get inactivated by bacterial antioxidant responses^10^. While host cytoplasmic NADPH oxidase is a primary producer of ROS, xanthine oxidoreductase can also participate in ROS production. Under normal conditions, the enzyme exists as xanthine dehydrogenase (XDH), which uses NAD^+^ as a cofactor to form NADPH during the enzymatic reactions to form xanthine and urate. This is the version of the enzyme predominantly detected in mollusks, arguing against a role for molluscan xanthine oxidoreductase in ROS production^11,12^. However, the mammalian XDH can be converted to xanthine oxidase (XO), which uses molecular oxygen as an electron acceptor instead of NAD^+^, thereby generating the ROS superoxide radical (O_2_^.−^) and hydrogen peroxide during the production of xanthine and urate^13^. As a result, bacterial infection in mammals may be associated with increased levels of these purines; for example, infection with enteropathogenic *Escherichia coli* resulted in intestinal accumulation of urate in a rabbit model of infection^14^. Furthermore, xanthine oxidoreductase can also use nitrate as substrate to form nitric oxide (NO) during hypoxic conditions^15^. Like ROS, NO and reactive nitrogen species derived from NO target cellular macromolecules to cause cell death unless detoxified by the bacterial cell.

Members of the multiple antibiotic resistance regulator (MarR) protein family are important for bacterial responses to environmental change^16^. MarR proteins are often autoregulatory and they respond to changing environmental cues such as small molecule ligands, oxidants, or pH, the result of which is a conformational change in the protein that alters (usually abrogates) DNA binding and changes expression of target genes. The genes encoding MarR family proteins are frequently oriented divergent to genes under their regulatory control, and binding sites for the transcription factor (usually 16–18 bp palindromes) are located in the intergenic region. One example is PecS, first characterized in the plant pathogen *Dickeya dadantii*^17^; the *pecS* gene is divergently oriented to *pecM*, which encodes a transporter responsible for efflux of the antioxidant indigoidine. Both genes are repressed by PecS binding to the *pecS-pecM* intergenic region. *D*. *dadantii pecS* knockout strains showed an upregulation of numerous virulence genes, including genes encoding pectinases and indigoidine biosynthetic genes^17,18^. While the inducing signal for *D*. *dadantii* PecS remains unknown, PecS proteins from *Agrobacterium fabrum*, *Streptomyces coelicolor*, *Klebsiella pneumoniae*, and *Pectobacterium atrosepticum* have been shown to bind urate. As a result of urate binding to PecS, DNA binding is attenuated and target genes are upregulated^19–23^. The accumulation of urate in mammalian and plant tissues during infection rationalizes why bacteria may use this purine metabolite as a signal for induction of virulence-associated genes^14,24^. For example, the urate-responsive transcription factor MftR from *Burkholderia thailandensis* controls numerous virulence-associated genes that are differentially expressed on addition of urate^25^. Divergent *pecS-pecM* gene pairs are only encoded by select bacterial species, including members of the gamma-proteobacterial families *Enterobacteriaceae* and *Vibrionaceae*^23^.

We show here that duplications of *pecS-pecM* genes are common in Vibrios. In *V*. *vulnificus*, which harbors a single *pecS-pecM* locus, PecS binds not only *pecS-pecM* intergenic DNA, a result of which is attenuated gene expression. It also controls activity of the promoter driving expression of nitric oxide dioxygenase, an enzyme that converts nitric oxide to nitrate to prevent nitrosative stress. Properties of *V*. *vulnificus* PecS are consistent with control of gene expression under conditions of host-mediated production of purine metabolites, such as molluscan hypoxia or production of ROS in mammalian hosts.

## Results

### Conservation of the *pecS-pecM* locus in members of the family ***Vibrionaceae***

The family *Vibrionaceae* comprises seven genera, *Aliivibrio*, *Echinimonas*, *Enterovibrio*, *Grimontia*, *Photobacterium*, *Salinivibrio*, and *Vibrio*, and numerous individual species have been identified within each genus. Many species are associated with marine environments, yet they are metabolically very diverse. A multilocus sequence analysis was performed in an effort to elucidate evolutionary history and genomic plasticity within the family *Vibrionaceae*; this analysis suggested the existence of 22 distinct clades plus two orphan clades and a superclade consisting of *Salinivibrio*, *Grimontia* and *Enterovibrio* spp^26^.

A BLASTp search of species representing the identified *Vibrio* clades using the *V*. *vulnificus* PecS (VvPecS) sequence was performed using the NCBI database. PecS homologs were confirmed by pair-wise alignment, and divergent genes were identified to assess the distribution of *pecS-pecM* gene pairs. VvPecS was previously shown to share significant homology to characterized PecS proteins from other bacterial species^23^; features distinguishing PecS proteins from canonical MarR homologs include a helical N-terminal extension (α1) containing a conserved tryptophan (Fig. S1)^23^. PecM proteins are characterized by two copies of the EamA protein domain (previously denoted DUF6) and are part of the drug/metabolite transporter (DMT) superfamily, as predicted by Pfam.

Divergent *pecS-pecM* genes were identified among many of the surveyed species, but not all. Specifically, divergent *pecS-pecM* genes were found in species belonging to the clades *Mediterranei*, *Scopthalmi*, and *Vulnificus* (to which *V*. *vulnificus* belongs) and in members of the *Salinivibrio-Grimontia-Enterovibrio* superclade (Table S1). Among other clades, only some species were found to encode *pecS-pecM* genes, and no *pecS* genes were identified in the clades *Damselae*, *Halioticoli*, *Porteresiae*, *Rosenbergii*, or in the orphan clade *Tapetis*. By contrast, some species belonging to the remaining clades were found to encode duplications of *pecS-pecM* gene pairs (*Cholerae*, *Coralliilyticus*, *Harveyi*, *Nereis*, *Orientalis*, and *Proteolyticus*). For some species belonging to the clades *Cholerae*, *Harveyi*, *Mediterranei*, *Nigripulchritudo*, and *Phosphoreum*, *pecS*-like genes were identified that were not divergently oriented to a *pecM* gene; in most cases, these species also encode *pecS-pecM* gene pairs.

**Figure 1:**
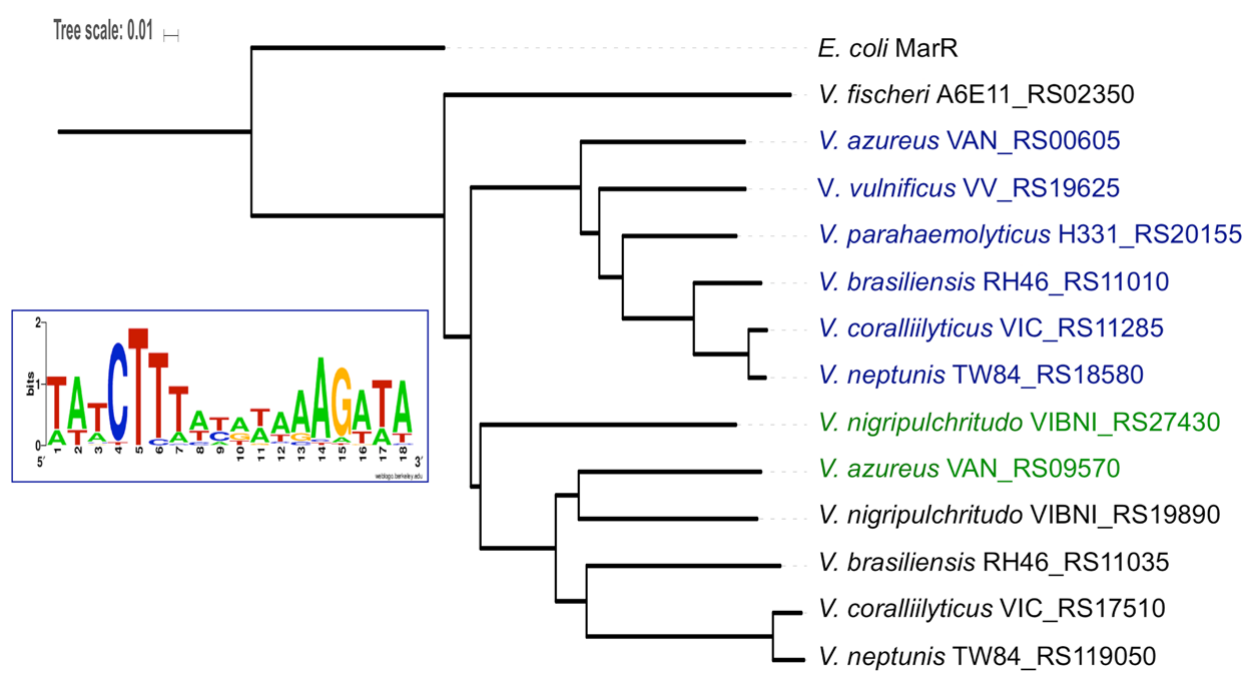
Duplication of PecS in some *Vibrio* species. Sequences of identified PecS proteins were aligned using MUSCLE and the phylogenetic tree was visualized with iTOL, using *E*. *coli* MarR as an outgroup. Sequences in blue representing the more conserved PecS sequences. Insert shows conservation of PecS binding sites in the intergenic region of conserved *pecS-pecM* gene pairs.

An alignment of select PecS sequences, representing species that encode a single *pecS-pecM* gene pair (such as *V*. *vulnificus*) as well as species with *pecS-pecM* duplications (such as the coral pathogen *V*. *coralliilyticus)* or *pecS* encoded in a different genomic environment (such as the shrimp pathogen *V*. *nigripulchritudo*) verified conservation of residues previously reported to be important for folding and/or interaction with the ligand urate (Fig. S1). For species with *pecS-pecM* duplications, one PecS shares more similarity to VvPecS (Fig. 1; species identified in blue) whereas the second PecS is less conserved (Fig. 1; shown in black). A phylogenetic analysis of selected PecS sequences using the neighbor-joining method indicated that the more divergent PecS sequences cluster on a distinct branch.

The intergenic *pecS-pecM* sequences corresponding to the more conserved PecS sequences were examined for the presence of PecS consensus binding sites^23^. All sequences were found to feature at least one, but more commonly two such cognate sites. Compiling 26 predicted sites from 14 intergenic sequences yielded the expected AT-rich consensus sequence characterized by the most significant conservation within the first 6 bp of each 9 bp half-site (Fig. 1; inset). Consistent with the presence of a consensus binding site, the sequence of the recognition helices (α5) is highly conserved (Fig. S1). The conservation of divergent *pecS-pecM* genes along with the cognate PecS binding site(s) strongly suggests conserved regulation of *pecS/pecM* expression by PecS, likely in response to similar signals.

### VvPecS binds *pecS-pecM* intergenic DNA

**Figure 2:**
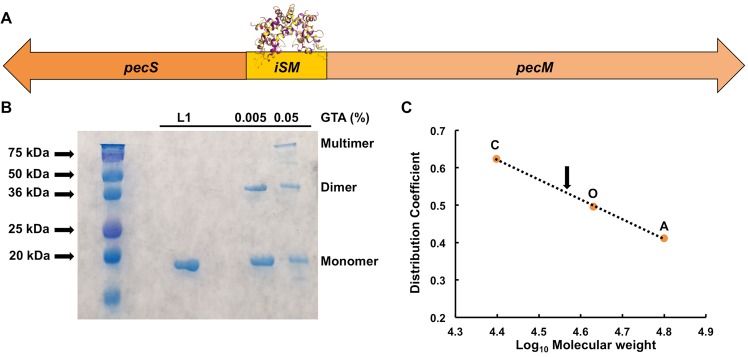
*V*. *vulnificus* PecS is a dimer. (**A**) Schematic representation of divergently oriented *V*. *vulnificus pecS* and *pecM* genes*;* VvPecS is expected to bind the intergenic DNA region *iSM*. (**B**) Coomassie Blue-stained 15% SDS-PAGE gel showing purified His_6_-tagged VvPecS (L1) and VvPecS incubated with 0.005% or 0.05% glutaraldehyde (GTA). Oligomeric states are indicated at the right. Mw markers (in kDa) at the left. (**C**) Size exclusion chromatography. Distribution coefficient as a function of Log_10_ Molecular weight of markers C, O, and A representing chymotrypsin (25 kDa), ovalbumin (44 kDa) and albumin (66 kDa), respectively. Elution of VvPecS marked with arrow. Uncropped version of gel in panel B shown in Fig. S7.

VvPecS is expected to control the expression of *pecS*-*pecM* genes by binding the intergenic DNA (denoted *iSM*; Fig. 2A). To address this prediction, the gene encoding VvPecS (*BJE04_RS19600*) was cloned from *V*. *vulnificus* YJ016 genomic DNA (a generous gift from G. Pettis), and the protein was overexpressed in *E*. *coli* and purified to ~95% homogeneity (Fig. 2B). Upon treating VvPecS with glutaraldehyde (0.005% GTA), the protein monomer (Mw ~18.6 kDa) crosslinked to form a dimer of ~37 kDa. Multimeric forms of VvPecS also appeared at a higher glutaraldehyde concentration. To confirm the oligomeric state of the purified protein, size exclusion chromatography was performed; VvPecS eluted corresponding to the molecular weight of a dimer (~37 kDa; Fig. 2C). The protein also migrated as a single band on native PAGE (Fig. S2), which is consistent with a single species in solution. In the following description of DNA binding, all concentrations refer to the dimeric form of VvPecS.

**Figure 3:**
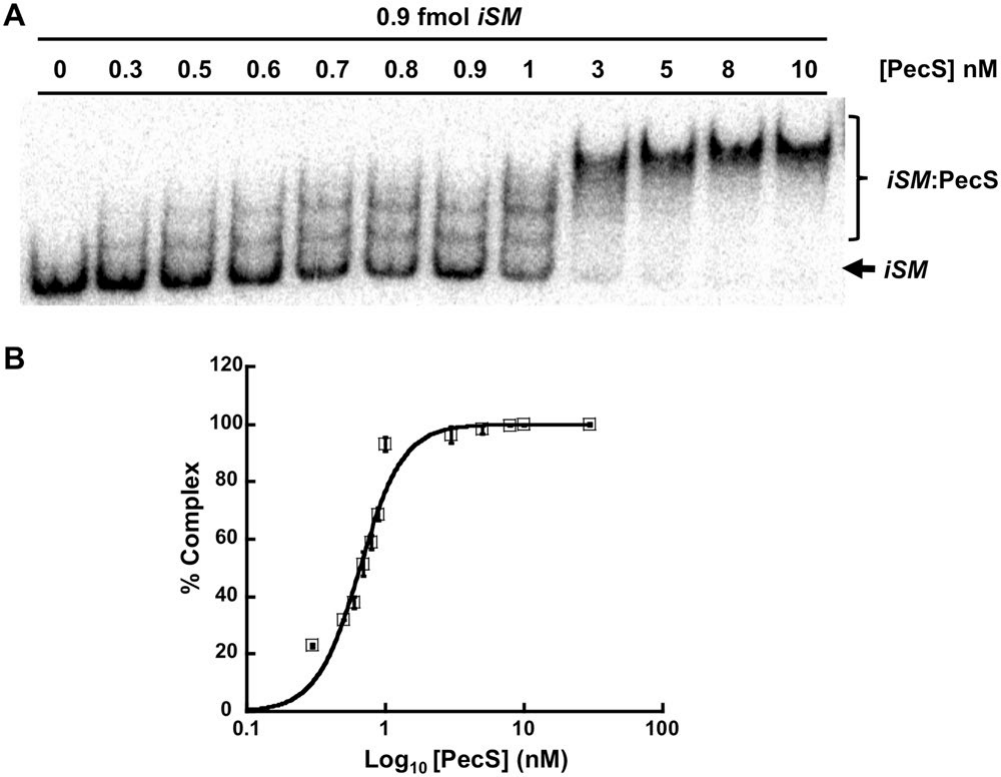
VvPecS binds multiple sites in the *pecS-pecM* intergenic region. (**A**) EMSA gel showing VvPecS binding to 0.9 fmol of intergenic *iSM* DNA; concentrations of PecS are identified above each lane (in nM). Complexes (*iSM*:PecS) and free DNA (*iSM*) are identified at the right. (**B**) Percent complex as a function of Log_10_ VvPecS concentration. *Error bars represents standard deviation (SD) of three replicates*. Uncropped version of gel in panel A shown in Fig. S8.

Binding to *pecS-pecM* intergenic DNA was assessed by the electrophoretic mobility assay (EMSA). Using the 200 bp *iSM*, which spans the region between *pecS* and *pecM* start codons, two distinct complexes were formed upon addition of lower concentrations of VvPecS (Fig. 3A), with additional complexes appearing with increasing VvPecS concentration; this suggests that the protein bound to multiple sites in the intergenic DNA. The percentage complex formation (total bound DNA) as a function of [PecS] was fitted to the Hill equation, which yielded an apparent macroscopic dissociation constant K_d_ = 0.3 ± 0.1 nM. Positive cooperativity of binding was indicated by a Hill coefficient n_H_ = 2.7 ± 0.1, also suggesting at least three binding sites on the *iSM* DNA fragment. The binding reactions were performed at high ionic strength (0.5 M Tris, 100 mM NaCl), which would disfavor nonspecific binding. Specificity of VvPecS binding to *iSM* DNA was verified by the retention of VvPecS-*iSM* complex when the reactions were challenged with increasing concentration of pUC18 plasmid (Fig. S3A).

**Figure 4:**
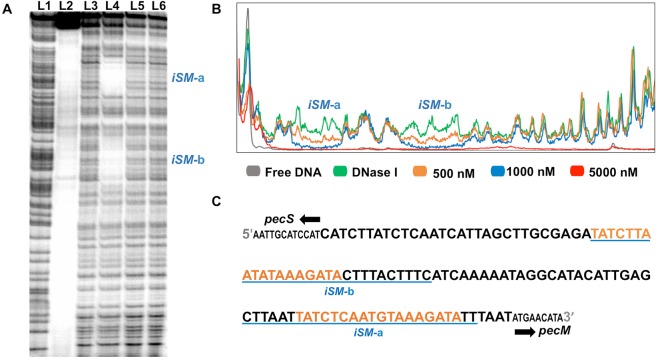
DNase I footprint of forward strand of *pecS*-*pecM* intergenic DNA. (**A**) Polyacrylamide sequencing gel: lane L1 – A + G ladder, L2 – undigested *iSM* fragment (500 nM); L3 – DNase I-digested *iSM* DNA; L4 to L6 – *iSM* DNA incubated with 1000 nM, 100 nM and 10 nM VvPecS, respectively, followed by DNase I digestion. (**B**) Densitometric traces of VvPecS protection of *iSM* (500 nM) DNA upon incubation with 0 nM (green), 500 nM (orange), 1,000 nM (blue) and 5,000 nM (red) VvPecS. C. Sequence of *iSM* intergenic sequence. The start codons of *pecS* and *pecM* are marked by solid black arrows. The pseudo-palindromic sequences are represented by orange bolded letters and protection by 1,000 nM PecS is underlined in blue. Reactions were performed under stoichiometric conditions ([DNA] ≫ K_d_). Data are representative of three experiments. Uncropped version of gel in panel A shown in Fig. S9.

VvPecS sites in the *iSM* DNA were mapped by DNase I footprinting. Using equivalent concentrations of DNA and VvPecS (each at 500 nM) a partial protection of two pseudo-palindromic sequences *iSM*-a and *iSM*-b was seen (Fig. 4B; orange line). At a stoichiometric excess of VvPecS:DNA of 2:1, complete protection of these sites was observed (Fig. 4A, lane 4, and 4B, blue line). Each protected site included the 18 bp consensus sequence and extended asymmetrically by about 8–10 bp (Fig. 4C). The *iSM*-a site is located 6 bp upstream of the *pecM* start codon, whereas *iSM*-b is 29 bp upstream of the *pecS* start codon, positions that would be consistent with repression of both genes by VvPecS. The two-fold excess of VvPecS required to protect *iSM* sites indicated that binding of two protein dimers occurs simultaneously and preferentially at the two palindromic sites. The intergenic region was entirely protected at 5000 nM of VvPecS (a 10-fold excess), with the region of protection extending well into the *pecS* coding region (Fig. 4B; red line). This is consistent with the positive cooperativity of binding seen in EMSA and with the inference that the *iSM* DNA can accommodate at least three PecS dimers. It also indicates that the multiple complexes observed in EMSA are due to VvPecS-DNA binding events as opposed to initial VvPecS-DNA complex formation followed by VvPecS-VvPecS binding.

### Xanthine and urate are ligands for VvPecS

**Figure 5:**
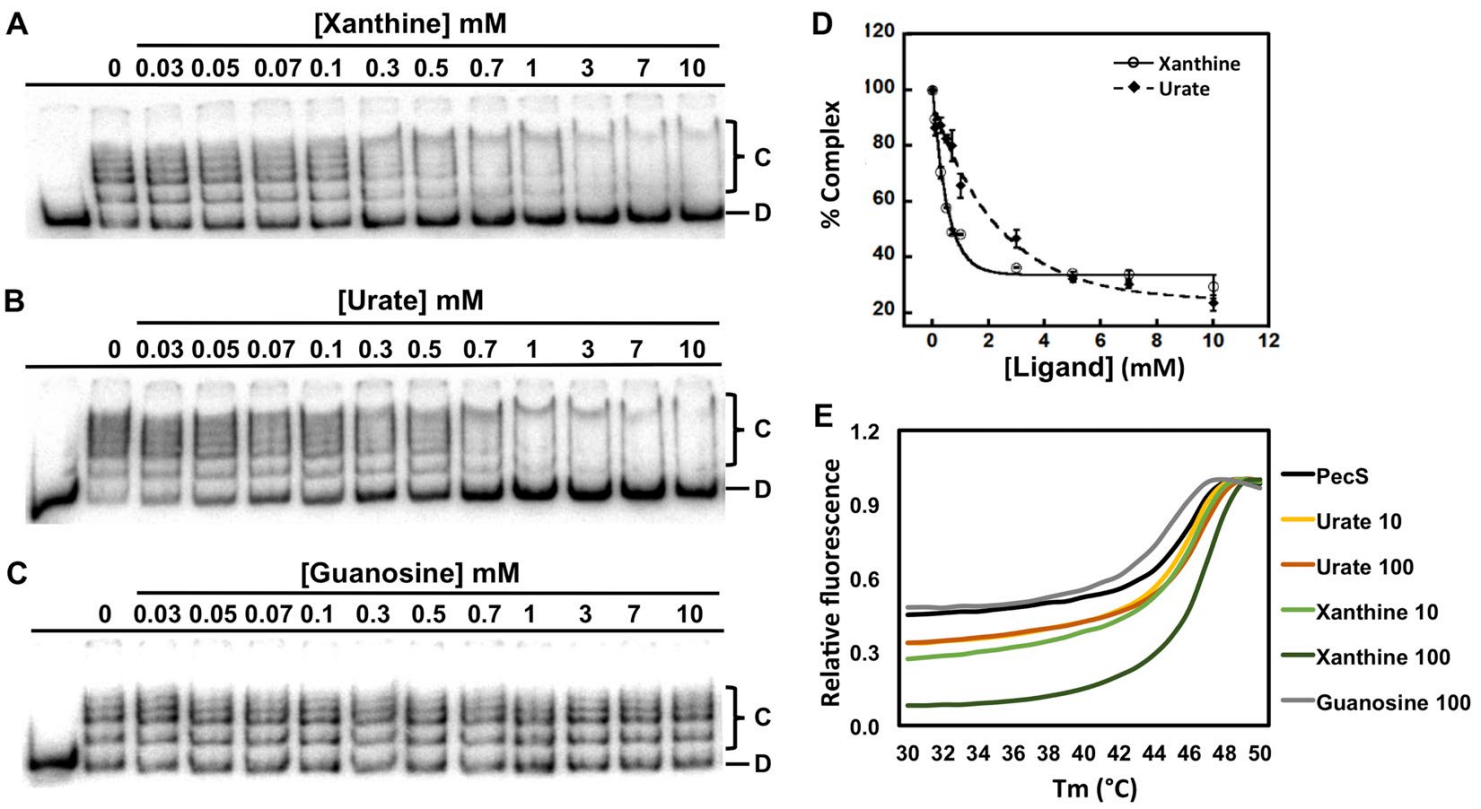
Xanthine and urate are ligands for VvPecS. A-C. EMSA gels showing titrations of VvPecS (0.8 nM) and *iSM* DNA (0.9 fmol) with increasing concentration of ligands xanthine (**A**), urate (**B**), and guanosine (**C**). Free DNA (“D”) and protein-DNA complexes (“C”) identified at the right and ligand concentrations indicated above each lane. (**D**) *Normalized percent complex formation as function of ligand concentration for urate and xanthine*. *Error bars represent standard deviation (SD) of three replicates*. (**E**) Thermal stability of VvPecS. Fluorescence of SYPRO Orange bound to exposed hydrophobic regions of the protein as a function of temperature; fluorescence is normalized to the peak fluorescence. Unliganded VvPecS (black), and VvPecS incubated with urate (10 µM in yellow and 100 µM in orange), xanthine (10 µM in light green and 100 µM in dark green) and guanosine (100 µM in grey). The data are average relative fluorescence from triplicates. Uncropped versions of gels in panels A–C shown in Figs S10–S12.

Several PecS homologs have been characterized and shown to bind urate, leading to attenuation of DNA binding^19–22^. Therefore, we tested whether increasing concentrations of intermediates in the purine degradation pathway such as urate and xanthine had an effect on VvPecS binding to *iSM* DNA (Fig. 5). The reactions contained 0.5 M Tris to negate the pH change that would otherwise be caused by the addition of ligands, which were dissolved in 0.4 M NaOH. Xanthine was found to attenuate PecS-*iSM* DNA binding with IC_50_ of 366** ± **31 µM (K_i_ = 60 ± 5 μM), which is ~4-fold better than urate for which IC_50_ was 1.5** ± **0.3 mM (K_i_ = 226 ± 29 μM) (Fig. 5A,B,D). However, even on addition of urate or xanthine up to 10 mM, the slowest-migrating VvPecS-*iSM* complex still remained, suggesting that neither ligand was able to disrupt a complex in which the DNA is saturated with protein; urate was modestly more efficient at disrupting this complex than xanthine (comparing basal plateaus in Fig. 5D). Guanosine and other intermediates in purine degradation, allantoin, hypoxanthine, GMP, and GTP did not affect DNA binding by VvPecS (Figs 5C and S4).

The interaction between ligands and VvPecS was further assessed with a thermal stability assay in which binding of the fluorescent dye SYPRO Orange to hydrophobic regions of the protein was monitored as a function of temperature. Unliganded VvPecS had a T_m_ of 45.0 ± 0.2 °C, indicating that the protein is stable at physiological temperatures. Both xanthine and urate very marginally stabilized VvPecS (T_m_ of 46.4 ± 0.2 °C and 46.0 ± 0.2 °C, respectively; Fig. 5E and Table S2). A more marked difference in the fluorescence profile that occurred on ligand (particularly xanthine) binding was the lower fluorescence at the beginning of the temperature scan, a change not observed on addition of guanosine; since fluorescence is a reporter for exposure of hydrophobic protein regions, this change may reflect burial of hydrophobic residues on ligand binding.

### VvPecS represses gene expression *in vivo*

**Figure 6:**
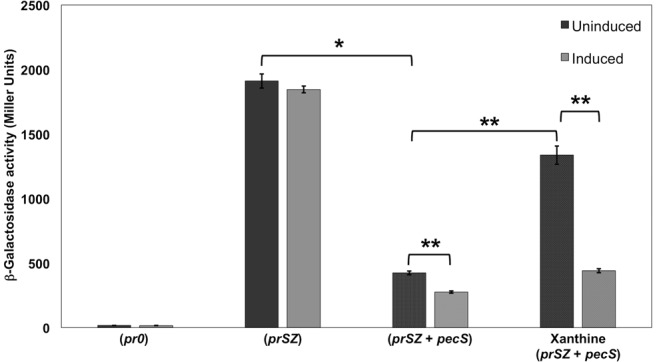
*In vivo lacZ* reporter gene expression assay. *E*. *coli* T7 Express was transformed with *pecS* expression construct and/or reporter construct: (*pr0*) – empty pACYC184 and pET28b; (*prSZ*) – *lacZ* under control of *pecS* promoter and empty pET28b; (*prSZ* + *pecS*) – *lacZ* under control of *pecS* promoter and *pecS* expression construct. Black bars represent uninduced cultures (no IPTG) and grey bars induced cultures (80 μM IPTG to induce *pecS* expression). Where indicated, xanthine was included at 10 mM concentration. β-galactosidase activity is represented in Miller units. Error bars represent the standard deviation from three replicates. Asterisks represent statistically significant difference based on two-tailed Student’s t-test (*p < 0.05 and **p < 0.005).

To examine the ability of VvPecS to repress gene activity *in vivo*, a reporter construct was generated in which the *lacZ* gene is under control of the *pecS* promoter (named *prSZ*). *E*. *coli* T7 Express was transformed with the reporter construct along with pET28b carrying the *pecS* gene or with empty pET28b. Compared to an empty pACYC184 negative control, significant β-galactosidase activity was observed for *prSZ* when cells were co-transformed with empty pET28b (Fig. 6, black bars); as expected, addition of IPTG had no effect on β-galactosidase activity (grey bars).

When *E*. *coli* cells were co-transformed with *prSZ* and the *pecS*-expression vector (*prSZ* + *pecS*), β-galactosidase activity was significantly reduced, regardless of whether or not IPTG was added to induce *pecS* expression, although a further reduction in activity was recorded in induced cells. Compared to cells harboring *prSZ* only, the β-galactosidase levels were ~4.5- and 7-fold lower in uninduced and induced cells producing VvPecS, respectively. This indicates that VvPecS represses the expression of the *IacZ* reporter, and that leaky (uninduced) expression is sufficient for repression (Fig. 6).

Since xanthine was an efficient ligand for VvPecS *in vitro* (and less prone to precipitation compared to urate) we determined its ability to induce *lacZ* expression; upon cellular uptake, the metabolite xanthine would be expected to undergo conversion to urate or to enter the purine salvage pathway, ultimately to generate GTP. Presence of xanthine in the growth medium resulted in significantly increased β-galactosidase activity in uninduced cells (~3-fold higher relative to untreated cells; Fig. 6). In cells to which IPTG was added to induce *pecS* expression, a modest 1.6-fold increase in β-galactosidase activity was observed on addition of xanthine. This indicates that xanthine (and/or urate) can relieve the VvPecS-mediated repression of the *lacZ* reporter, except when PecS is overexpressed as a result of IPTG induction.

### VvPecS binds the intergenic region between *nsrR* and *nod* genes

To identify other possible genes under VvPecS control, Patloc was used to search the *V*. *vulnificus* YJ016 genome using the perfectly palindromic VvPecS site 5′-TATCTTTATATTAAGATA-3′ as a query, allowing for five mismatches^27^. Sequences upstream of annotated genes in which the mismatches primarily occur within the last 3 bp of each half-site (the least-conserved part of the overall PecS consensus sequence; Fig. 1) were examined. Several potential sites within gene promoters were identified on both chromosomes I and II, including sites in the intergenic region between *BJE04_RS14760* and *BJE04_RS14765* encoding the transcription factor NsrR and a nitric oxide dioxygenase (NOD), respectively. NsrR encodes a nitric oxide sensitive response regulator, which upon NO-mediated damage to its iron-sulfur cluster no longer binds DNA. This leads to derepression of genes encoding proteins involved in nitric oxide detoxification, such as *nod*. Nitric oxide dioxygenase converts NO to nitrate in a reaction that requires oxygen^28^.

A single palindromic site 5′-TTTCTTGAT·TGAAAGATA-3′ was found 35 bp upstream of the start codon of the *nsrR* gene in the intergenic DNA (*iNR*) region of divergently oriented *nsrR*-*nod* genes (Fig. S5A). This site is also conserved in the intergenic region between *V*. *parahaemolyticus nsrR* and *nod* genes (*VP2808* and *VP2809*; not shown). VvPecS bound the *iNR* fragment (Fig. S5B) with a K_d_ of 218 ± 38 nM (Fig. S5D), a considerably lower affinity compared to the K_d_ of VvPecS for *iSM*. Specificity of VvPecS-*iNR* binding was indicated by the retention of complex upon addition of an up to ~5,000-fold excess of nonspecific lambda DNA (48.5 Kbp) (Fig. S5C). In contrast, the complex gradually disappeared when titrated with up to ~90-fold excess of unlabeled *iNR* (Fig. S5C). This indicates that the binding of VvPecS to the single site in *iNR* is specific. The VvPecS-*iNR* complex was attenuated in the presence of xanthine and urate, with IC_50_ of 242 ± 69 µM (K_i_ = 73 ± 21 µM) and 744 ± 96 µM (K_i_ = 226 ± 29 µM), respectively (Fig. S6). The attenuation of VvPecS-*iNR* complex in the presence of ligands follows a similar trend as that of VvPecS-*iSM*, with xanthine and urate functioning as ligands.

### VvPecS mediates repression of *nod* promoter activity *in vivo*

**Figure 7:**
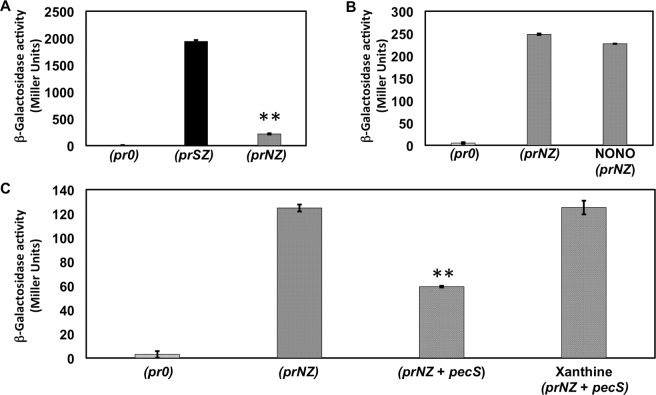
VvPecS represses *nod* promoter activity *in vivo*. *In vivo lacZ* reporter gene assay in which *E*. *coli* T7 Express was transformed with *pecS* expression construct and/or reporter construct. β-galactosidase levels (in Miller units) in uninduced *E*. *coli* expression host harboring gene constructs: (*pr0*) – empty pACYC184 and pET28b; (*prSZ*) – *lacZ* under control of *pecS* promoter and empty pET28b; (*prNZ*) – *lacZ* under control of *nod* promoter and empty pET28b; (*prNZ* + *pecS*) – *lacZ* under control of *nod* promoter and *pecS* expression construct. (**A**) Comparison between *prSZ* and *prNZ* promoter activities in the absence of VvPecS (asterisks indicate *p* < 0.005). (**B**) Activity of *prNZ* in absence or presence of NONOate (25 μM). (**C**) Effects of VvPecS and xanthine on activity of *prNZ*. Asterisks indicate *p* < 0.005 compared to (*prNZ*) cells based on two-tailed Student’s t-test. Error bars represents standard deviation from three replicates.

A reporter construct was generated in which *lacZ* is under control of the *nod* promoter (named *prNZ*) and β-galactosidase activity was measured in *E*. *coli*. In absence of the *pecS* expression vector, an ~9-fold lower β-galactosidase activity was measured for *prNZ* compared to *prSZ*, suggesting that the *nod* promoter is weaker than the *pecS* promoter in *E*. *coli* (Fig. 7A). Due to the lower levels of *lacZ* expression in the *prNZ* construct, uninduced conditions (leaky expression of *pecS*) were maintained for assessing the ability of VvPecS to repress *nod* promoter activity.

Examination of the *iNR* sequence also revealed a possible binding site for *E*. *coli* NsrR^29^ that could interfere with the expression analysis. To test if there was any influence of the endogenous *E*. *coli* NsrR on the *prNZ* activity, 25 µM of the NO donor spermine NONOate was added. Expression of the reporter gene was not significantly affected by addition of NONOate, arguing against inhibition of gene activity by endogenous *E*. *coli* NsrR, as addition of NO would otherwise have been expected to increase gene activity (Fig. 7B).

In cells transformed with both *prNZ* and the *pecS* expression construct (*prNZ* + *pecS*), the leaky production of PecS resulted in repression of *lacZ* expression by ~2-fold compared to cell cultures harboring only the *prNZ* construct (Fig. 7C). Addition of xanthine to the culture medium restored β-galactosidase activity to that observed in cells not expressing *pecS* (Fig. 7C). Taken together, these observations indicate that VvPecS represses the *nod* promoter and that xanthine and/or urate relieves this repression.

## Discussion

PecS family proteins are classified by a combination of sequence conservation and structural features as well as being part of a genomic locus that includes the divergently encoded PecM^18–22^. PecS consistently functions as a repressor of *pecS/pecM* genes by binding to the *pecS-pecM* intergenic DNA, and VvPecS conserves this function. PecS paralogs conserve the characteristic structural features, but they are not encoded divergently from a *pecM* gene, suggesting acquisition of a distinct regulatory function. For example, *Streptomyces* spp. encode both PecS and TamR, a transcription factor that regulates genes encoding enzymes involved in central metabolism during oxidative stress^20,30,31^. TamR does not respond to urate, but instead to citrate and structurally related compounds. PecS paralogs (of unknown function) were also detected in a few *Vibrio* genomes (Table S1). The existence of *pecS-pecM* duplications in Vibrios is more intriguing, as similar duplications have not been reported in other species. It raises the possibility of distinct responses that have been optimized for the lifestyle of particular species, for example during stress adaptation, either in terms of inducing signal, control of cellular PecS concentration, or control of cellular function by modulation of the PecS regulon^32^. One possibility is the acquisition of a direct response to oxidants; while VvPecS and closely related PecS proteins have no cysteine residues, the second group of PecS homologs features two conserved cysteines (in the loop between α1 and α2 and immediately preceding β3; Fig. S1). The sequence of the DNA recognition helix (α5; Fig. S1) is also more divergent in the second group of PecS homologs, perhaps allowing recognition of different cognate DNA sites and therefore defining a distinct regulon.

VvPecS binds two specific AT-rich palindromic sites in *pecS-pecM* intergenic DNA (Fig. 4), with additional flanking sites bound at higher protein concentrations, whereas a single complex was detected in its interaction with *nsrR-nod* intergenic DNA (Fig. S5). By comparison, *D*. *dadantii* PecS protects extended regions in target gene promoters, but no distinct palindromic sequences were identifiable^33^. In contrast, *A*. *fabrum* PecS binds to three palindromic sites, one in the *pecS* promoter and two overlapping sites in the *pecM* promoter, and *P*. *atrosepticum* PecS protects two discrete sites in *pecS-pecM* intergenic DNA^19,22^. Protection of extended DNA regions at higher protein concentrations may ensure more efficient repression of gene activity when PecS is in excess.

**Figure 8:**
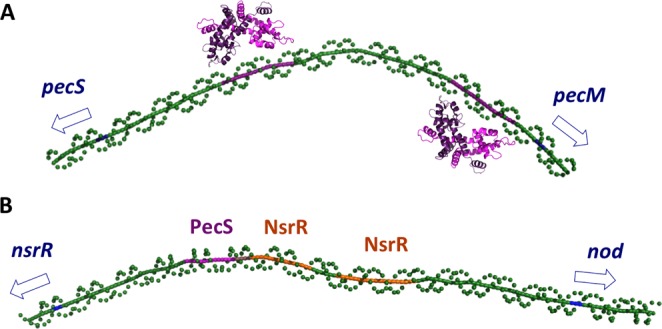
Gene regulation by VvPecS. A. Predicted curvature of *iSM* DNA calculated based on the dinucleotide wedge model using https://www.lfd.uci.edu/~gohlke/dnacurve/. The distance between centers of palindromes predicts binding of VvPecS (magenta) to opposite faces of the duplex. Palindromic binding sites for PecS in magenta. Translational starts identified in blue and with an open arrow. B. Predicted global conformation of *iNR* DNA with VvPecS site in magenta and predicted NsrR sites in orange; the PecS and NsrR sites overlap by 4 bp. A site that resembles the *E*. *coli* NsrR consensus binding site (AANATGCATTT) overlaps the predicted *V*. *vulnificus* NsrR site in the *nod* promoter^29^.

A global 3D model of the *pecS-pecM* intergenic DNA calculated based on the dinucleotide wedge model suggests the existence of significant curvature, a feature that would be expected to promote association with RNA polymerase (reflecting greater promoter strength; Fig. 8)^34^. Sites for VvPecS are located on either side of the apex; based on the distance between the centers of palindromes (57 bp), a binding mode in which the two first-bound VvPecS dimers are bound on opposite faces of the helix is predicted.

In addition to sites in *pecS-pecM* intergenic DNA, VvPecS binds to a conserved palindromic site between *nsrR* and *nod* genes, but with lower affinity. The lower affinity binding implies that a greater cellular concentration of PecS would be required for repression. Consistent with this inference, repression of *pecS* promoter activity is more efficient than repression of the *nod* promoter (Figs 6 and 7). *In vivo*, elevated levels of PecS would be expected in the wake of a burst of PecS ligand, allowing maximal expression of *pecS*. Since the ligands (urate and xanthine) are naturally occurring metabolites that would be degraded or salvaged, such conditions would then be predicted to lead to excess PecS accumulating on the *pecS* promoter to ensure efficient repression as well as repression of *nsrR/nod* expression. A similar site in *nsrR-nod* intergenic DNA is found in *V*. *parahaemolyticus* (another well-known human pathogen causing gastroenteritis following consumption of raw oyster), suggesting that PecS-mediated control of *nod* gene expression is conserved.

Host cells produce ROS and reactive nitrogen species (RNS) upon infection to kill invading bacteria. While NADPH oxidase is the primary source of ROS, XO can contribute to host defenses by using molecular oxygen to generate O_2_^.−^/H_2_O_2_ (under normoxia) and using nitrate to generate NO (under hypoxia), in the process generating xanthine and urate. In mollusks, however, only certain species (stenoxic bivalves, which tolerate only a narrow range of oxygen levels) have been reported to exhibit XO activity, with XDH activity being responsible for the accumulation of urate that characterizes hypoxia^9,12,13^. The significance of these purine metabolites is that they accumulate under stress, either in infected vascular tissues of the human host or during molluscan hypoxia. This accumulation rationalizes why *Vibrio* spp. would recognize these stress markers as signals to induce expression of genes required to survive the adverse conditions.

VvPecS conserves the amino acids previously identified as required for urate binding and for communicating ligand binding to the DNA recognition helices (Fig. S1). *In vitro*, addition of ligand to *pecS-pecM* DNA that was saturated with PecS did not result in complete dissociation of PecS, but retention of a slow-migrating complex corresponding to PecS-saturated DNA (Fig. 5). This could be an artifact of the *in vitro* condition where high PecS concentration was used. However, if cellular levels of PecS were highly elevated, retention of promoter-bound PecS even in presence of ligand would prevent complete derepression of *pecS* and ward off further PecS synthesis until cellular PecS levels return to a normal range. Consistent with this interpretation, addition of xanthine to cells expressing PecS significantly increases activity of the *pecS* promoter, but not to the level of cells devoid of PecS, reflecting residual repression (Fig. 6). While this may in part derive from the degradation or salvage of xanthine that would be expected to occur upon cellular uptake, thereby lowering the effective concentration of ligand, it may also reflect the failure of ligands to disrupt a complex in which *pecS* promoter DNA is saturated with PecS. An argument in favor of the latter interpretation is that addition of xanthine fully restores *nod* promoter activity to the level observed in absence of PecS; only a single PecS-DNA complex was observed on *nod* promoter DNA (Figs S5 and 7).

The reported accumulation of urate during molluscan hypoxia and in infected mammalian tissues identifies situations in which VvPecS ligands would be abundant. Our data also suggest a unique target for VvPecS, namely control of the gene encoding NOD. NsrR from several bacterial species has been shown to regulate the gene encoding the flavohemoglobin nitric oxide dioxygenase (the *nod* gene is denoted as *hmp* in many species) and other genes that are involved in detoxification of NO and in repair of nitrosothiol species^28,35^. NsrR contains an iron-sulfur cluster, which is stable in the presence of oxygen. These [Fe-S] clusters are susceptible to NO, a result of which is derepression of genes under NsrR control.

Despite its toxicity, NO may also serve signaling functions in beneficial interactions. Regulatory networks appear to vary among *Vibrio* species, with NsrR identified as the primary NO-sensing regulator of *hmp* (*nod*) gene activity in *V*. *fischeri*, while the *V*. *cholerae hmp* gene is thought to be primarily controlled by the activator NorR^36^. Consistent with this inference, binding sites for NsrR are predicted in several target genes according to RegPrecise^37^, including in *V*. *fischeri*, *V*. *parahaemolyticus*, and *V*. *vulnificus*, while none are predicted in *V*. *cholerae*. Notably, two binding sites for *V*. *vulnificus* NsrR are predicted in the *nsrR-nod* intergenic region, one of which overlaps the PecS site (Fig. 8B). This suggests that binding of NsrR and VvPecS would be mutually exclusive. Repression of *nod* promoter activity by VvPecS suggests a complex regulatory mechanism in which maximal *nod* expression is achieved under conditions of NO production as well as absence of PecS binding as a result of either presence of PecS ligand or tight control of *pecS* expression; since molluscan hypoxia is characterized by urate accumulation as well as the ability of XDH to produce NO, this may be one such scenario.

NO is also part of the initial “oxidative burst” generated by eukaryotic cells as an antimicrobial tactic, and it may react with other compounds to form reactive nitrogen species. Inducible nitric oxide synthase (iNOS) can generate large quantities of NO during the mammalian inflammatory process^38^, and the bacterial NOD is therefore critical for detoxification of NO^36^. In the low-oxygen environment of the intestinal tract colonized by Vibrios, NOD can convert NO to nitrate (in an oxygen-requiring process) or to nitrous oxide (anaerobically). This is also an environment in which XO-mediated production of ROS has been shown to result in accumulation of urate^14^. NO damages the NsrR iron-sulfur cluster, a result of which is derepression of *nod*, and a simultaneous ligand-binding to VvPecS will ensure maximal *nod* expression.

In the continued absence of VvPecS ligand, VvPecS would control *pecS* expression and maintain VvPecS homeostasis, and cellular levels would likely be insufficient for binding to the *nod* gene promoter, which would remain under control of NsrR. However, when VvPecS ligands accumulate (e.g., during molluscan hypoxia), upregulation of *pecS* would occur, leading to increased cellular levels of VvPecS. As the VvPecS ligands are metabolized (degraded or salvaged), cellular levels of VvPecS may then be sufficient for repression of *nod* expression. While mammalian tissues experience ischemic reperfusion injury during recovery from hypoxia due to ROS production by XO, the ability of mollusks to tolerate anoxic to normoxic transitions have been correlated with absence of XO activity^12^. During prolonged anoxia, NO levels have been reported to decrease and to remain low in some tissues even 12 h after reoxygenation^39^; in these scenarios, VvPecS-mediated repression of *nod* may be required to avert depletion of NO.

## Material and Methods

### Sequence analysis and protein modeling

Amino acid sequences were aligned using MUSCLE^40^. The phylogenetic tree was generated using Clustal Omega and visualized with iTOL, using *E*. *coli* MarR as an outgroup^41^. A VvPecS model was generated with SwissModel in automated mode using the structure of HucR (2fbk) as template and used to map secondary structure elements in the sequence alignment^42^.

### Cloning and purification of VvPecS

The genomic DNA of *V*. *vulnificus* YJ016 (provided by Gregg Pettis, LSU) was used as a template to amplify the gene encoding VvPecS (locus tag BJE04_RS19600; old locus tags VVA0819 and VV_RS19625). Primers S1 and S2 containing NdeI and EcoRI restriction sites were used to amplify the *pecS* gene (for sequences, see Table S3). The resulting PCR product was cloned into the pET28b expression vector at the NdeI-EcoRI sites and the construct was verified by sequencing.

The verified construct was transformed into *E*. *coli* BL21(DE3)pLysS. The cells were grown at 37 °C in Luria Bertani (LB) broth with 50 µg/mL kanamycin until OD_600_ reached ~0.7. Overexpression of VvPecS was achieved by inducing the culture with 1 mM isopropyl β-D-1-thiogalactopyranoside (IPTG) for 3 hours at 37 °C. The cells were pelleted by centrifugation and stored at −80 °C. The cell pellets were thawed on ice and ~1 g was resuspended in 1 mL of Resuspension (R) buffer (50 mM sodium phosphate pH 7.0, 200 mM NaCl, 0.15 mM PMSF (phenylmethylsulfonyl fluoride) and 1% (v/v) glycerol). A final concentration of 2 mg mL^−1^ of lysozyme was added to the cell suspension and cells were lysed on ice for 1 hr with intermittent vortexing and sonication. The lysate was collected by centrifugation at 10,000 × g for 30 min and incubated with nickel beads (Sigma-Aldrich, His-Select® Nickel Affinity Gel) that were pre-washed and equilibrated in R buffer at 4 °C for 1 hr. The beads were collected by centrifugation at low speed and washed twice with 10 volumes of R buffer and once with 10 volumes of R buffer containing 10 mM imidazole. The beads were transferred into a gravity flow column and the protein was eluted with R buffer containing a linear gradient of imidazole ranging from 20 mM to 200 mM. The purified protein eluates were pooled and concentrated using Amicon® Ultra Filters and buffer exchanged with R buffer containing 5% glycerol. The purified protein was stored at −80 °C in aliquots and was verified to be ~95% pure on Coomassie Blue G-250 stained SDS-PAGE gels. The VvPecS protein concentration was calculated based on the absorbance at 280 nm and an extinction coefficient of 15470 cm^−1^ M^−1^ (VvPecS contains two tryptophan and three tyrosine residues per monomer).

A glutaraldehyde crosslinking assay was performed by incubating 1 µl of 0.005 and 0.05% glutaraldehyde (GTA) with 2 µl of 33 µM VvPecS in a 5 µl reaction using R buffer for 15 minutes at 25 °C. The reaction was stopped by addition of 5 µl 2X Laemmli buffer without β-mercaptoethanol. The samples were separated on 15% SDS-PAGE gels and visualized by Coomassie Blue G-250 staining.

### Size exclusion chromatography

Size exclusion chromatography was performed using 50 mM phosphate buffer, pH 7.5, with 150 mM NaCl and a Superdex 200 10/300 GL (GE Healthcare) column. The elution volumes (V_e_) for marker proteins chymotrypsin (25 kDa), ovalbumin (42.7 kDa), and albumin (66 kDa) were used to calculate the distribution coefficient using the equation (V_e_ − V_o_)**/**(V_t_ − V_o_), with geometric bed volume, V_t_ and void volume, V_o_. The distribution coefficient versus Log_10_ of Molecular weight of markers was used to extrapolate the molecular weight of VvPecS.

### Thermal stability assay

VvPecS (2 µM) was incubated in an assay buffer (50 mM Tris pH 8.0, 50 mM NaCl) with 5X SYPRO Orange (Invitrogen) reference fluorescent dye in the presence or absence of ligands urate, xanthine, or guanosine. Fluorescence emission was measured at a range of temperatures (0 to 94 °C) in 1 °C increments for 45 s in an Applied Biosystems 7500 real-time PCR system. A negative control without protein was included to correct the fluorescence yield. The melting curve obtained was fit to a four-parameter sigmoidal equation using Sigma Plot 9 to calculate the (T_m_) melting temperature of VvPecS. Experiments were performed in triplicate and the Tm reported as mean ± SD.

### Electrophoretic mobility shift assay (EMSA) and quantification of protein-DNA complexes

The respective intergenic DNA regions *iSM* and *iNR* between *pecS-pecM* (*BJE04_RS19600-19605*) and *nod-nsrR* (*BJE04_RS14765-RS14760*; old locus tags *VV3064-3063* and *VV_RS14775-14770*) genes were amplified using the primers listed in Table S3; for *iSM*, the template was *V*. *vulnificus* YJ016 genomic DNA, and for *iNR* the template was plasmid pCR2.1 containing the synthesized *iNR* sequence. The DNA fragments were radiolabeled at their 5′ ends using γ-[^32^P] ATP and T4 polynucleotide kinase. The protein-DNA binding affinity was determined using EMSA. A 10 µl reaction containing 1 fmol of [^32^P]-labeled intergenic DNA and increasing concentration of purified VvPecS was incubated at 25 °C for 20 minutes in binding (B) buffer (0.5 M Tris pH 8.0, 100 mM NaCl, 6% (v/v) glycerol, 25 µM EDTA, 0.12% Brij-58 and 50 µg of bovine serum albumin). The reaction mixtures were electrophoresed using 0.5 X Tris-borate-EDTA buffer on 8% polyacrylamide gels (39:1 (w/w) acrylamide: bisacrylamide) at 170 V for 1.5 hr at 25 °C; gels were pre-run for 15 min prior to loading the samples. The gel was dried and exposed to a storage phosphor screen and scanned with a Typhoon 8600.

ImageQuant 5.1 (GE Healthcare) was used for quantification of complex and free DNA; total bound DNA was considered as complex and the region on the gel between complex and free DNA was also considered as complex to account for complex dissociation during electrophoresis. KaleidaGraph 4.0 (Synergy Software) was used for fitting of the data to f = f_max_ * [PecS]^n^/(K_d_ + [PecS]^n^) where [PecS] is the VvPecS protein concentration, f is fractional saturation, K_d_ is the apparent equilibrium dissociation constant and n is the Hill coefficient. K_d_ values are reported as the average ± the standard deviation of at least three replicates.

The specificity of VvPecS binding to the intergenic DNA was assessed by mixing [^32^P]-labeled *iSM* and *iNR* with a constant concentration of VvPecS and increasing concentration of non-specific DNA such as pUC18 and linearized Lambda DNA in B buffer.

The effect of ligands xanthine, urate, guanosine, GMP, hypoxanthine and allantoin on VvPecS DNA binding was determined by incubating the protein with the respective ligand at increasing concentration for 10 minutes in B buffer, followed by a 20 min incubation with DNA. The B buffer contains 0.5 M Tris pH 8.0 to prevent a pH change as the ligands were dissolved in 0.4 N NaOH. Reaction mixtures were electrophoresed and processed as described above. All experiments were performed in triplicate. The concentration of ligand, which inhibited 50% of the complex formation (IC_50_) was calculated using the equation *f = A + B X e*^*−k[L]*^, where *f* is fractional saturation, *k* is the decay constant, *[L]* is ligand concentration, *A* is the saturation plateau and *B* is the decay amplitude. The IC_50_ was converted to inhibition constants (K_i_) using a web-based tool (https://botdb-abcc.ncifcrf.gov/toxin/kiConverter.jsp), where K_i_ = IC_50_/([DNA]_50_/K_d_ + [PecS]_0_/K_d_ + 1), [DNA]_50_ is the concentration of DNA at 50% inhibition and [PecS]_0_ is the protein concentration at 0% inhibition^43^.

### DNase I footprinting

Forward primer S1 (Table S3) was radiolabeled and used to amplify the 200 bp *iSM* region for footprinting experiments to determine the sequence to which VvPecS binds. Footprinting reactions containing 500 nM *iSM* DNA with increasing concentration of VvPecS were incubated at 25 °C for 30 minutes in a binding buffer containing 20 mM Tris (pH 8.0), 50 mM NaCl, 0.06% (v/v) BRIJ58, 20 μg mL^−1^ BSA, 1.5% (v/v) glycerol 5 mM MgCl_2_, and 2.5 mM CaCl_2_. Twenty units of DNaseI was added and the samples were incubated for 30 s. The reaction was terminated by addition of an equal volume of loading dye (90% formamide, 10 mM EDTA, 0.1% bromophenol blue and xylene cyanol, 1 mM NaOH). The A + G ladder was prepared using a standardized protocol by Sambrook and Russell^44^. Samples were heated at 90 °C for 5 min prior to electrophoresis on an 8% denaturing gel [19:1 (w/w) acrylamide/bis-acrylamide, 8 M urea, and 1 × Tris–borate–EDTA buffer (pH 8.3)]. The gel was pre-run at ∼1800 V to reach ∼53 °C. The voltage was then adjusted to run samples at a constant temperature of 48 °C. Finally, the gel was dried at 80 °C under vacuum and exposed to a phosphor-imaging screen. The experiment was performed in triplicate.

### *In vivo* gene regulation

The gene expression analyses were performed using the *lacZ* reporter gene, expressed in plasmid pACYC184 under control of either the *pecS* promoter or the *nod* promoter. The *pecS-pecM* intergenic fragment flanked by BglII sites was amplified with primers S5 and S6 (Table S3) and *V*. *vulnificus* YJ016 genomic DNA as template and cloned into the unique BglII site upstream of *lacZ* in plasmid pRADZ1; only the orientation in which *lacZ* is under control of the *pecS* promoter was obtained. The *pecS* promoter-*lacZ* fragment was then subcloned into pACYC184 using NruI and BamHI to generate pACYC-*prSZ*. A promoter-less version of the pACYC-*prSZ* was obtained by BglII digestion to generate pACYC-*Z*. A synthetic construct was purchased in which the *nod-nsrR* intergenic fragment was flanked by BglII sites in pCR2.1, and the *nod-nsrR* fragment was subcloned into pACYC-*Z* at the BglII site to generate pACYC-*prNZ*; the orientation in which *lacZ* is under control of the nod promoter was obtained. The plasmid constructs were confirmed by sequencing. For both constructs, only one orientation of the intergenic DNA was obtained, even though this was a non-directional cloning. Since β-galactosidase is toxic to *E*. *coli* when exported to the periplasm^45^, we speculate that the opposite orientations of the insert may have introduced a sequence that was translated and interpreted as a signal sequence.

For analysis of gene regulation, plasmids pACYC-*prSZ* or pACYC-*prNZ* and the expression vector pET28b-*pecS* were co-transformed into *E*. *coli* T7 Express (which lacks endogenous β-galactosidase activity; New England BioLabs). Cells carrying the pACYC184 and pET28b plasmids were used as negative control, and cells carrying only pACYC-*prSZ* or pACYC-*prNZ* were used as positive control. Cells were grown in LB with chloramphenicol (100 µg mL^−1^; to select for pACYC184 derivatives) and kanamycin (50 µg mL^−1^; for pET28b derivatives) at 37 °C to 0.6–0.8 OD_600nm_ with or without IPTG (80 µM) and xanthine (10 mM). Cells expressing *lacZ* under control of the *nod* promoter were also treated with 25 µM spermidine NONOate to test for potential interference from *E*. *coli* NsrR. The cell processing and measurement of β-galactosidase activity using ONPG (*o*-nitrophenyl *β*-D-galactopyranoside) as substrate was performed as described^46^. The activity (in Miller units) was calculated using the equation 1000 × (A_420_ − 1.75 × A_550_)/(t × V × A_600_], where t is reaction time in minutes and V is volume. The culture set up and expression assays were repeated three times, each in technical triplicates.

## Supplementary information

Supplemental Information

## Data Availability

Materials are available from the corresponding author upon request.
