# Supplementary material for: A role for Vibrio vulnificus PecS during hypoxia
Source: Sci Rep. 2019 Feb 26;9:2797. doi: 10.1038/s41598-019-39095-4 (PMC6391409; doi:10.1038/s41598-019-39095-4)
Supplement: Supplementary file 1 — Supplemental Information [file 41598_2019_39095_MOESM1_ESM.pdf]

A role for *Vibrio vulnificus* PecS during hypoxia

Nabanita Bhattacharyya, Tiffany L. Lemon, Anne Grove
Department of Biological Sciences, Louisiana State University, Baton Rouge, Louisiana 70803

Contents

Figures:

Figure S1. Conservation of PecS sequences.

Figure S2. Migration of VvPecS on native PAGE gel.

Figure S3. Binding of VvPecS is specific.

Figure S4. Purine intermediates that do not affect DNA binding by VvPecS.

Figure S5. VvPecS binds the *nsrR-nod* intergenic region.

Figure S6. Xanthine and urate disrupt VvPecS-*iNR* complex formation.

Tables:

Table S1. *pecS-pecM* genes in *Vibrio* species.

Table S2. Thermal Stability of VvPecS in presence of ligands.

Table S3. Sequences of primers used for amplification, sequencing and creation of plasmid constructs.

Uncropped gels (all consisting of contiguous lanes, no splicing):

Figure S7. Panel 2B.

Figure S8. Panel 3A.

Figure S9. Panel 4A.

Figure S10. Panel 5A.

Figure S11. Panel 5B.

Figure S12. Panel 5C.

Figure S13. Panel S3A.

Figure S14. Panel S3B.

Figure S15. Panel S5B.

Figure S16. Panel S5C.

Figure S17. Panel S6A.

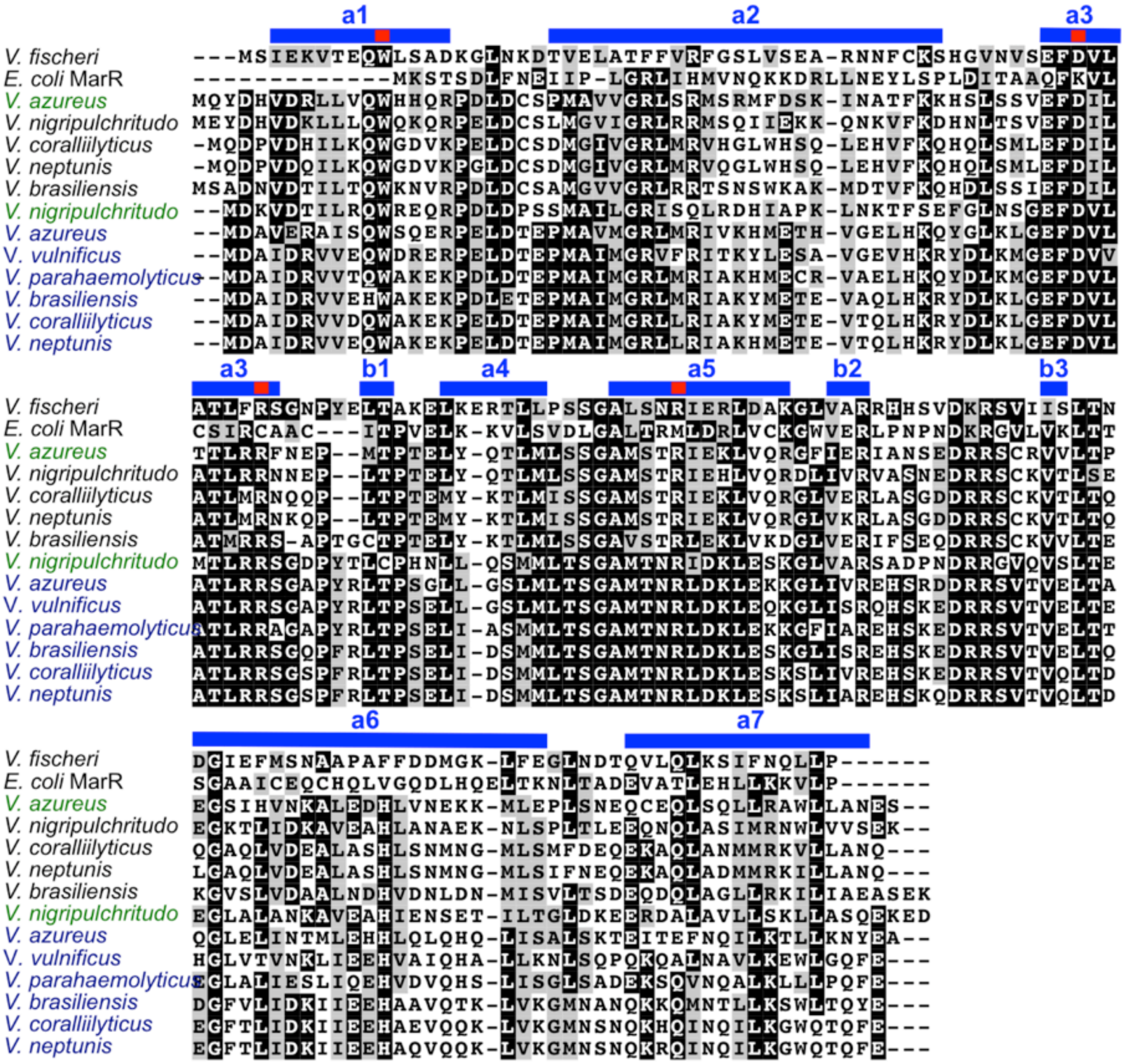

Figure S1. Conservation of PecS sequences. Sequences in blue represent the more conserved PecS sequences, sequences in green represent PecS encoded in a genomic context that does not include *pecM*, and sequences in black include examples of more divergent PecS sequences from strains in which the *pecS-pecM* locus is duplicated. Predicted secondary structure elements shown above the alignment are based on the structure of HucR (2fbk) (1). Red squares identify residues that are diagnostic of PecS proteins. Helix $\alpha5$ is the DNA recognition helix.

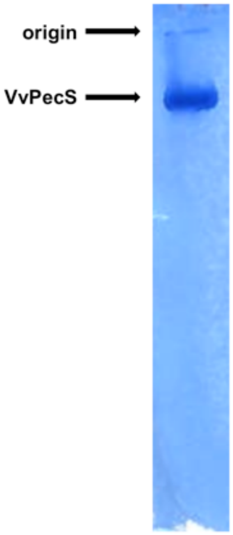

Figure S2. Migration of VvPecS on native PAGE gel. Migration of VvPecS (pI~6.5) on 8% native PAGE gel, pH 8.8. Entire gel is shown (not cropped lengthwise).

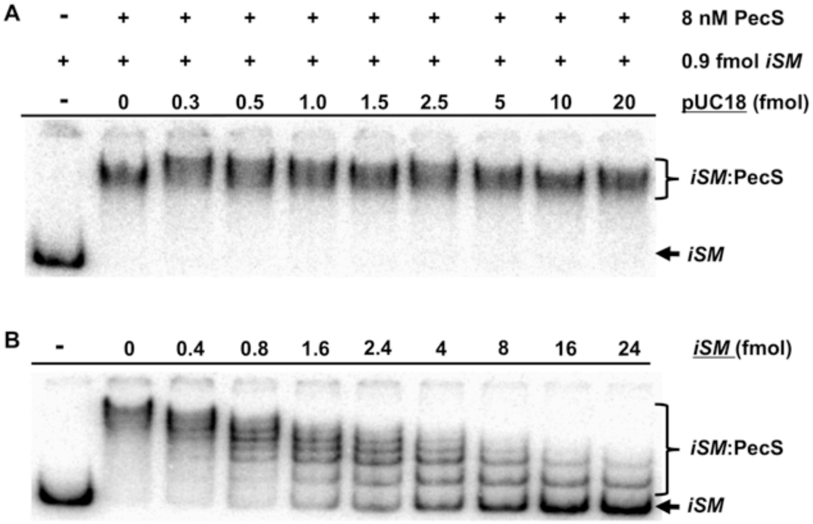

Figure S3. Binding of VvPecS is specific. VvPecS (8 nM) and *iSM* DNA (0.9 fmol) challenged with increasing concentrations of plasmid pUC18 (A) or unlabeled *iSM* DNA (B). Amount of competitor added is identified above each lane. Complexes (*iSM*:PecS) and free DNA (*iSM*) are identified at the right. Addition of 20 fmol pUC18 (2,686 bp) reflects a >250-fold excess of non-specific DNA relative to labeled *iSM*. By comparison, disappearance of the VvPecS-*iSM* complexes upon titration with up to 24-fold excess unlabeled *iSM* DNA confirmed specific binding. Uncropped versions of gels in panels A and B shown in Figures S13 and S14.

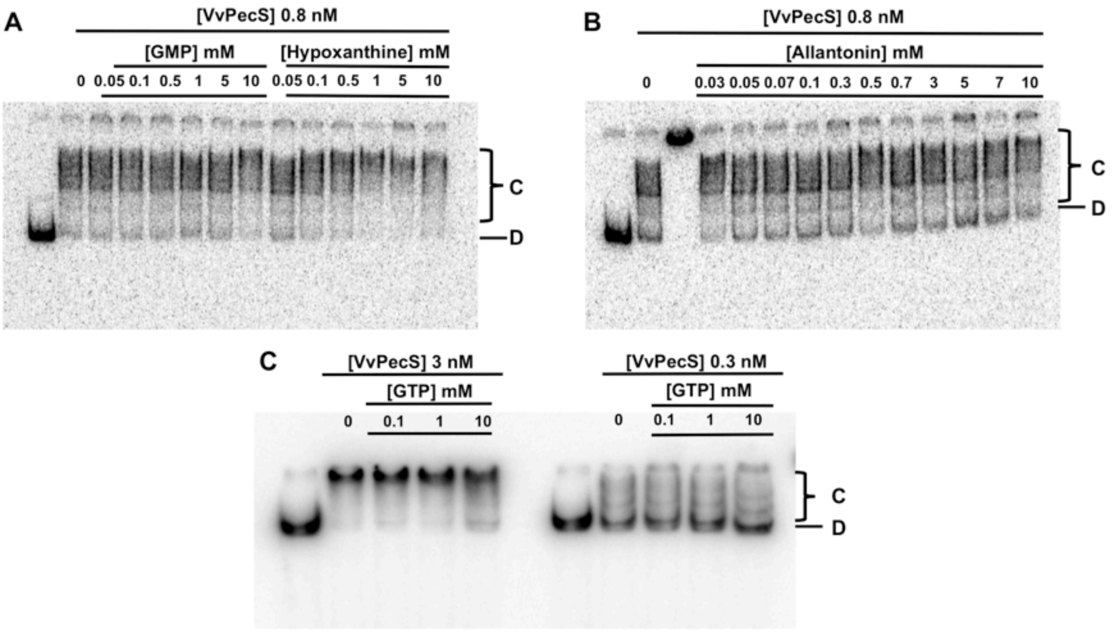

Figure S4. Purine intermediates that do not affect DNA binding by VvPecS. A-B. EMSA gels showing titrations of VvPecS (0.8 nM) and *iSM* DNA (0.9 fmol) with increasing concentration of GMP or hypoxanthine (A) or allantoin (B). C. EMSA gels showing titrations of VvPecS (3 nM or 0.3 nM) with increasing concentrations of GTP. Free DNA ("D") and protein-DNA complexes ("C") identified at the right and ligand concentrations indicated above each lane. The origins of all gels are shown (no cropping).

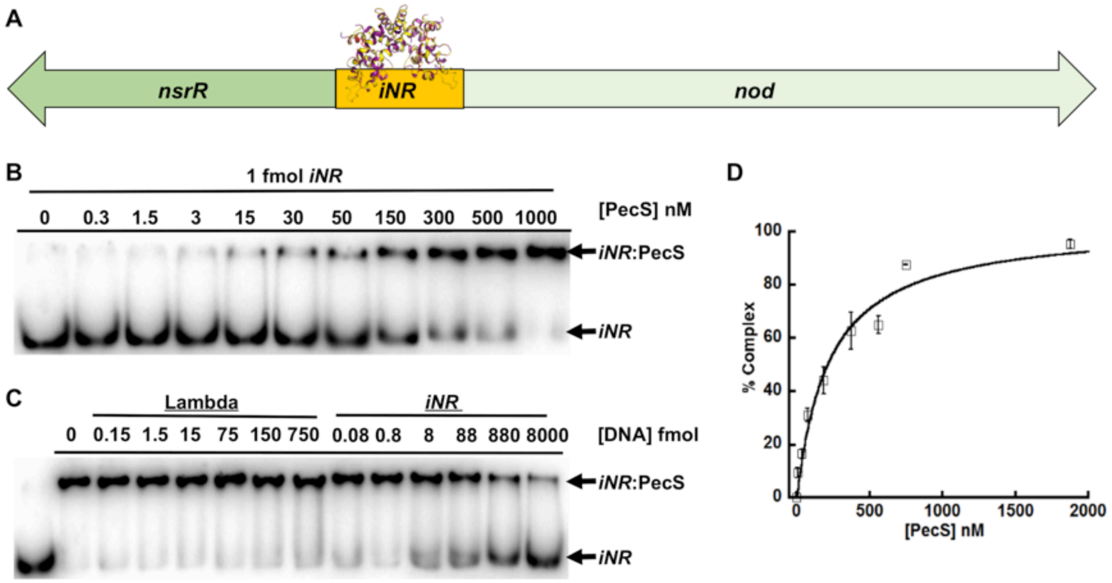

Figure S5. VvPecS binds the *nsrR-nod* intergenic region. A. Genomic locus encoding nitric oxide-sensitive repressor (*nsrR*) and nitric oxide dioxygenase (*nod*). The intergenic region between the two genes, *iNR,* has a probable binding site for VvPecS. B. EMSA gel showing binding of VvPecS to 1 fmol of *iNR*. Concentrations of VvPecS are identified above each lane. C. EMSA gel showing specificity of VvPecS binding to *iNR* DNA by titrating with unlabeled non-specific (lambda) DNA and specific DNA (*iNR*); amount of competitor added is indicated above each lane. The complexes (*iNR*:PecS) and free DNA (*iNR*) are identified at the right. D. Percentage complex formation as a function of VvPecS concentration. Error bars represent the standard deviation from three replicates. Uncropped versions of gels in panels B and C shown in Figures S15 and S16.

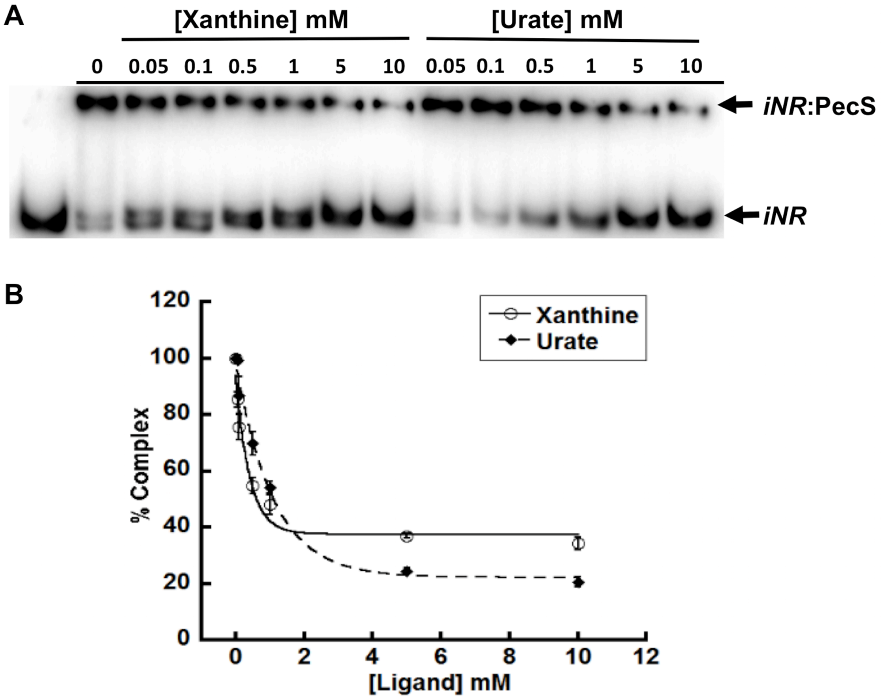

Figure S6. Xanthine and urate disrupt VvPecS-*iNR* complex formation. A. EMSA gel showing the effect of ligands xanthine and urate on VvPecS-*iNR* complex formation. Ligand concentrations are identified above each lane. B. Normalized percent complex formation as function of ligand concentration. Error bars represent the standard deviation from three replicates. Uncropped version of gel in panel A shown in Figure S17.

Table S1. ***pecS-pecM*** **genes in** ***Vibrio*** **species**

**Clade**	**Species encoding *pecS-pecM* genes**	**Species not encoding *pecS-pecM***	**Species with *pecS-pecM* duplication**	**Species with *pecS* in other genomic context**
Anguillarum	*V. anguillarum, V. ordalii*	*V. aestuarianus*		
Cholerae	*V. cholerae*<br>*V. mimicus*	*V. cincinnatiensis*	*V. furnissii*<br>*V. fluvialis*	*V. cholera, V. parilis,*<br>*V. metschnikovii*<br>*V. mimicus*
Coralliilyticus			*V. coralliilyticus*<br>*V. neptunis*	
Damselae		*P. damselae*		
Diazotrophicus	*V. diazotrophicus*	*V. hispanicus*		
Fischeri	*A. fischeri, A. logei*	*A. salmonicida*<br>*A. sifiae*<br>*A. vodanis*		
Gazogenes	*V. rhizosphaerae*	*V. aerogenes*<br>*V. gazogenes*<br>*V. ruber*		
Halioticoli		*V. breoganii*<br>*V. ezurae*<br>*V. gallicus*<br>*V. halioticoli*<br>*V. inusitatus*<br>*V. neonatus*<br>*V. superstes*		
Harveyi	*V. azureus*<br>*V. campbellii*<br>*V. communis*<br>*V. harveyi*<br>*V. parahaemolyticus*<br>*V. rotiferianus*		*V. alginolyticus*<br>*V. mytili*<br>*V. natriegens*	*V. azureus*<br>*V. communis*<br>*V. harveyi*
Mediterranei	*V. mediterranei*<br>*V. maritimus*<br>*V. variabilis*			*V. mediterranei*
Nereis	*V. xuii*		*V. nereis*	
Nigripulchritudo	*V. nigripulchritudo*	*V. penaeicida*		*V. nigripulchritudo*
Orientalis	*V. sinaloensis*		*V. brasiliensis*<br>*V. hepatarius*<br>*V. orientalis*<br>*V. tubiashii*	
Pectenicida	*V. cariibeanicus*	*V. pectenicida*		
Phosphoreum		*P. iliopiscarium*<br>*P. leiognathi*<br>*P. phosphoreum*		*P. angustum*
Porteresiae		*V. porteresiae*<br>*V. tritonius*		
Profundum	*P. profundum*	*P. indicum*<br>*P. lipolyticum*		
Proteolyticus			*V. proteolyticus*	
Rosenbergii		*P. lutimaris*<br>*P. rosenbergii*		
Rumoiensis	*V. rumoiensis*	*V. litoralis*		
Scopthalmi	*V. ichthyoenteri, V.*			

	ponticus<br>*V. scopthalmi*			
Splendidus	*V. crassostrea*<br>*V. cyclitrophicus*<br>*V. fortis*<br>*V. kanaloae*<br>*V. lentus*<br>*V. splendidus*<br>*V. tasmaniensis*	*V. chagasii*<br>*V. gigantis*<br>*V. pelagius*<br>*V. pomeroyi*		
Tapetis		*V. tapetis*		
Vulnificus	*V. navarrensis*<br>*V. vulnificus*			
Salinivibrio -Grimontia-Enterovibrio	*S. costocola,*<br>*G. hollisae,*<br>*E. coralii, E. norvegicus*			

Surveyed species belonging to identified clades (2). Species identified as encoding *pecS-pecM* gene pairs encode one such gene pair, while other species encode duplications of the divergent *pecS-pecM* genes. Species identified as not encoding *pecS-pecM* genes encode no *pecS*. Some species encode *pecS* in a different genomic environment; for *V. communis* and *V. harveyi*, a gene encoding a short hypothetical protein with no match in the Pfam database is encoded between *pecS* and *pecM* genes. In *P. angustum*, a gene encoding a predicted uracil phosphoribosyltransferase is inserted between *pecS* and *pecM* genes. Remaining species in this category do not encode *pecM* in the vicinity of the *pecS* gene.

Table S2. Thermal Stability of VvPecS in presence of ligands

	T_m (°C)[a]
VvPecS	45.0 ± 0.2
Xanthine 10 µM	45.3 ± 0.3
Xanthine 100 µM	46.4 ± 0.2
Urate 10 µM	45.1 ± 0.2
Urate 100 µM	46.0 ± 0.2
Guanosine 100 µM	44.0 ± 0.2

[a]Average T_m ± SD of three replicates

Table S3. Sequences of primers used for amplification, sequencing and creation of plasmid constructs

Gene inserts and Plasmids	Primer name, sequence and description
Intergenic DNA region	
iSM	S1: 5’-CTCGGTATCTAACTCTGGTCGC-3’<br>S2: 5’-GCCAACGTTGCTTGTGTAAC-3’<br>Amplicon size: 200 bp
iNR	N1: 5’-CTGGTGGGCTCCTCTATGGTGAAG<br>N2: 5’-GATCTAGCGGGCTTCCTATTAGGATTATTTGT-3’<br>Amplicon size: 154 bp
Primers with restriction site for DNA cloning	
pecS cloning	S3: 5’-GATAAG**CATATG**GATGCAATTGATC-3’<br>S4: 5’-GCGAC**GAATTC**GCTAAGTAATG-3’<br>NdeI and EcoRI restriction sites in bold
prSM cloning	S5: 5’-GTCACG**AGATCT**GCTTTACTTTCATC-3’<br>S6: 5’-GCTTAG**AGATCT**ACTCAATGTATGCC-3’<br>BglII restriction sites in bold<br>Amplicon size: 110 bp

Uncropped gels

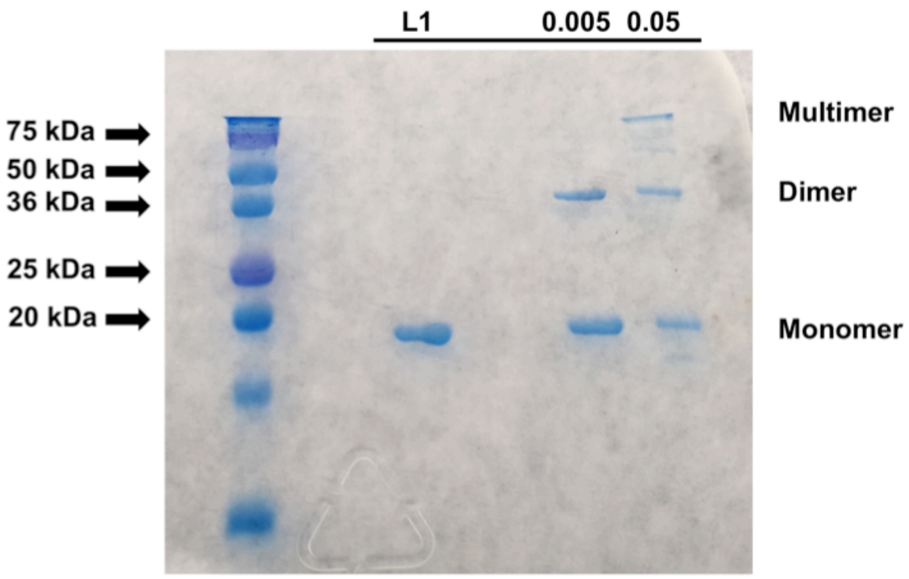

Figure S7. Panel 2B.

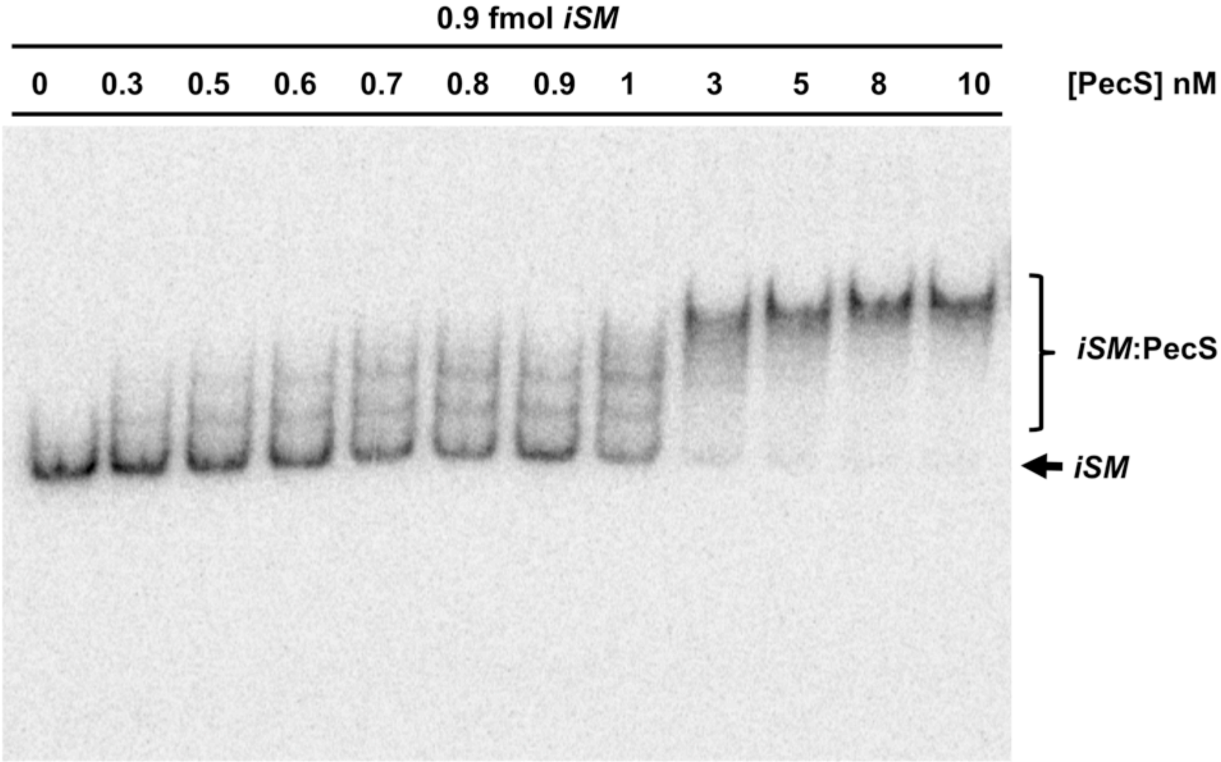

Figure S8. Panel 3A.

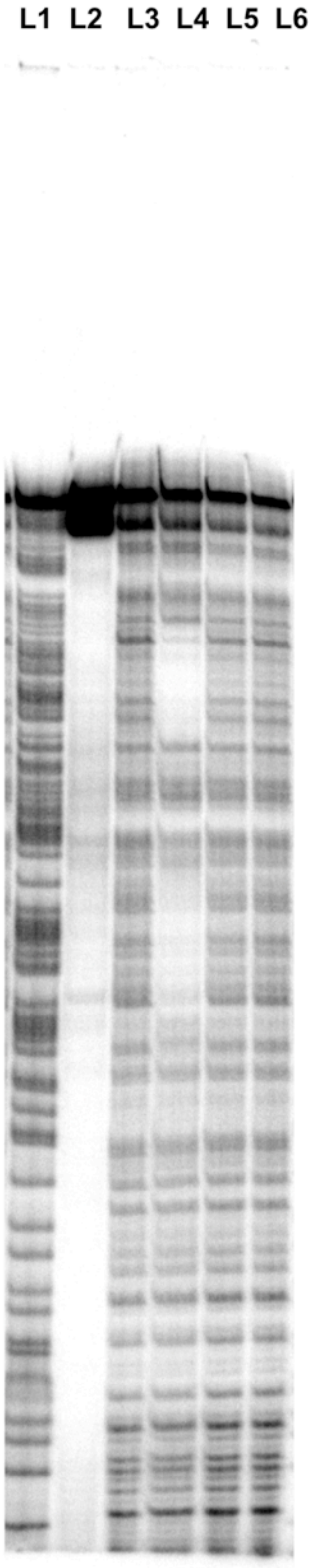

Figure S9. Panel 4A.

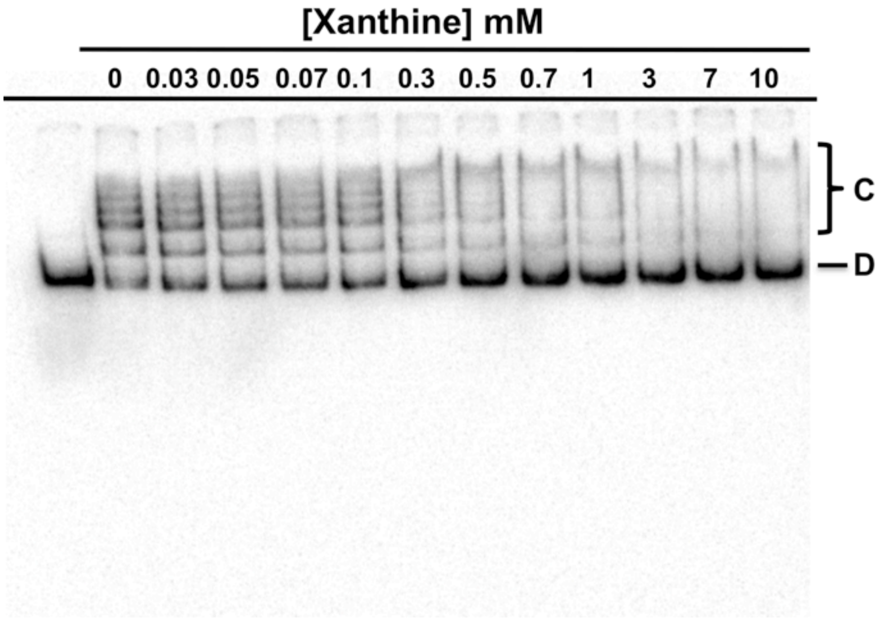

Figure S10. Panel 5A.

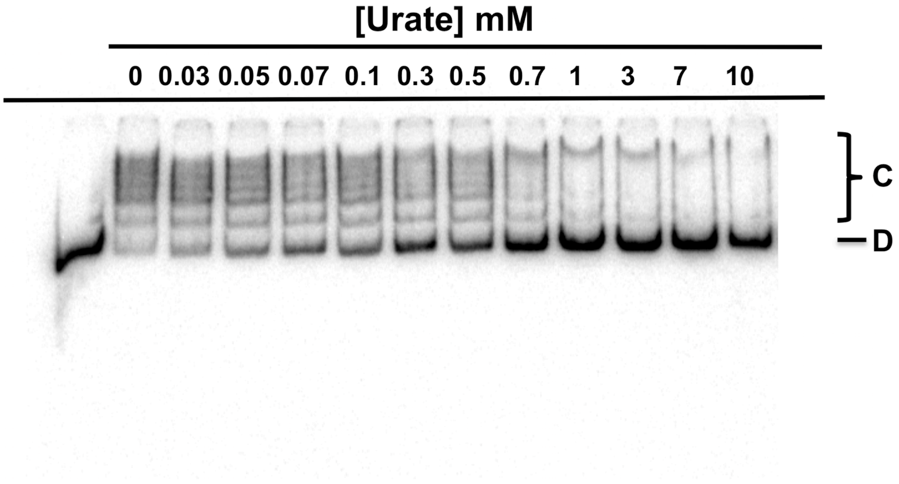

Figure S11. Panel 5B.

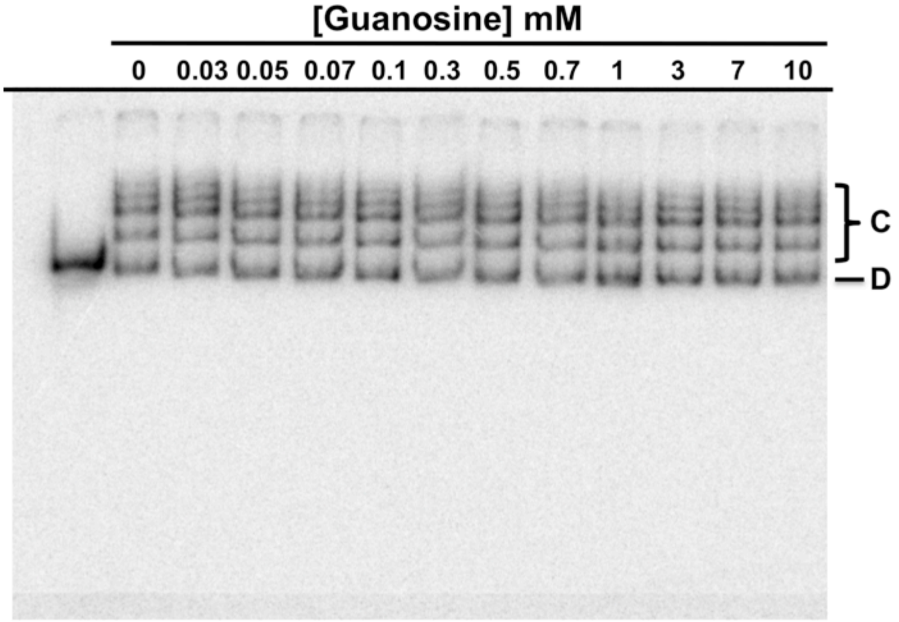

Figure S12. Panel 5C.

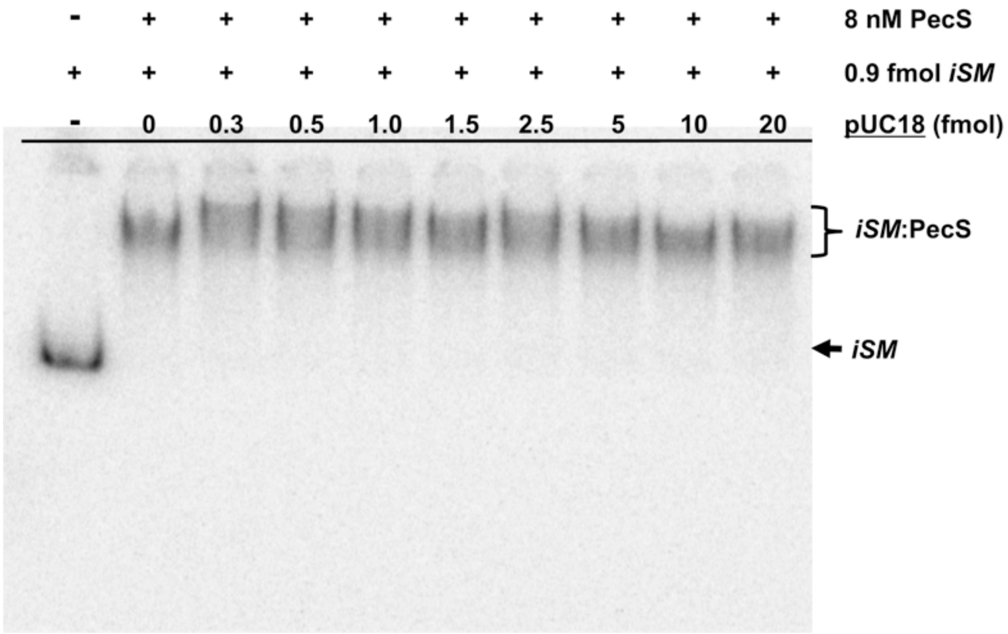

Figure S13. Panel S3A.

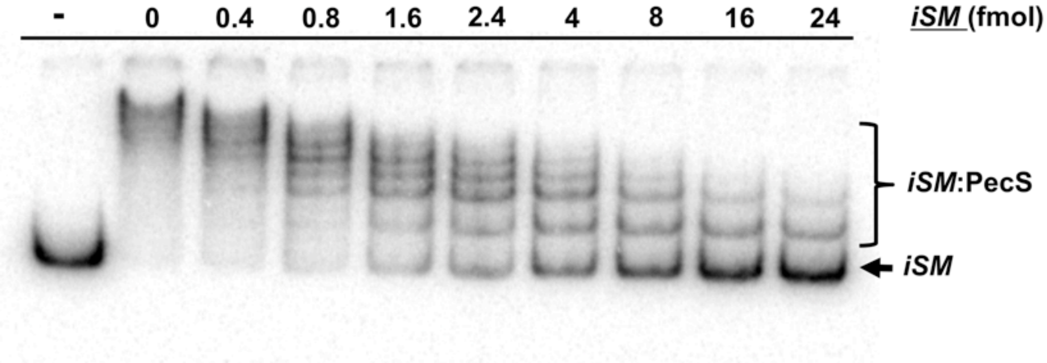

Figure S14. Panel S3B.

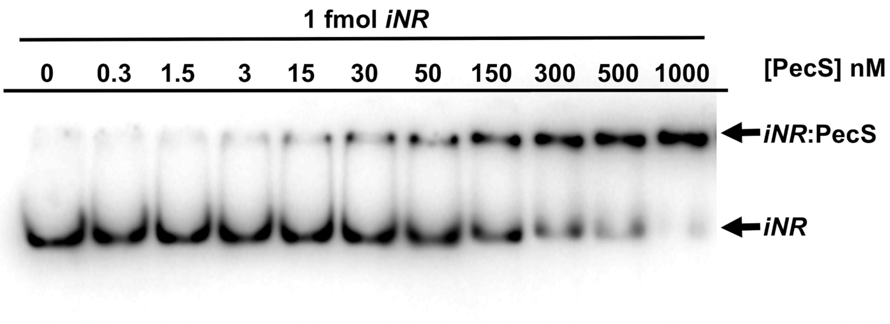

Figure S15. Panel S5B.

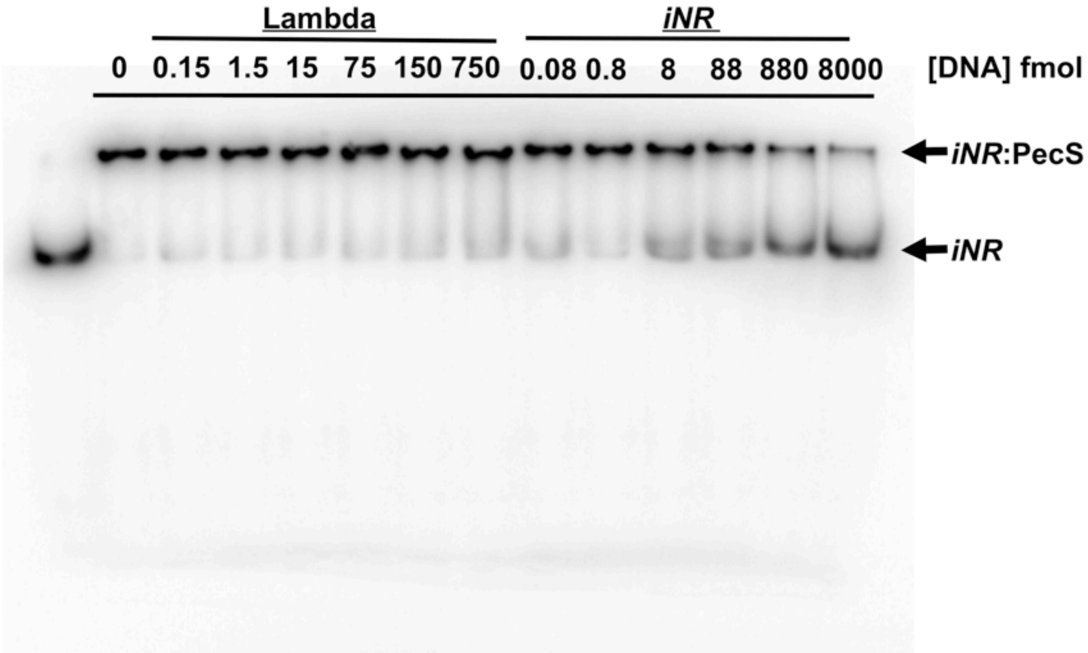

Figure S16. Panel S5C.

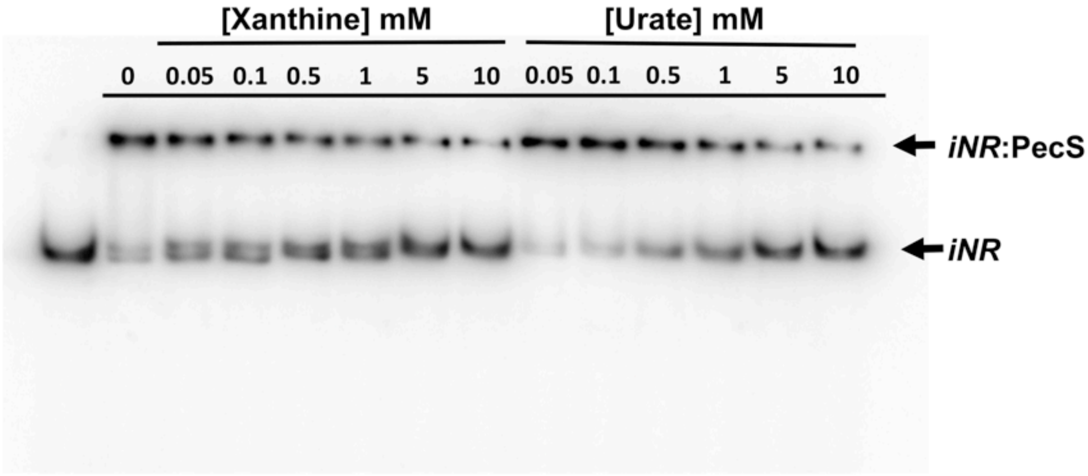

Figure S17. Panel S6A.

References

1. Bordelon, T., Wilkinson, S. P., Grove, A., and Newcomer, M. E. (2006) The crystal structure of the transcriptional regulator HucR from *Deinococcus radiodurans* reveals a repressor preconfigured for DNA binding. *J Mol Biol* **360**, 168-177
2. Sawabe, T., Ogura, Y., Matsumura, Y., Feng, G., Amin, A. R., Mino, S., Nakagawa, S., Sawabe, T., Kumar, R., Fukui, Y., Satomi, M., Matsushima, R., Thompson, F. L., Gomez-Gil, B., Christen, R., Maruyama, F., Kurokawa, K., and Hayashi, T. (2013) Updating the *Vibrio* clades defined by multilocus sequence phylogeny: proposal of eight new clades, and the description of *Vibrio tritonius* sp. nov. *Front Microbiol* **4**, 414
